# ET_A_ receptor activation contributes to T cell accumulation in the kidney following ischemia‐reperfusion injury

**DOI:** 10.14814/phy2.13865

**Published:** 2018-09-10

**Authors:** Erika I. Boesen

**Affiliations:** ^1^ Department of Cellular and Integrative Physiology University of Nebraska Medical Center Omaha Nebraska

**Keywords:** Endothelins, ET_A_ receptors, ischemia, kidney, T lymphocyte

## Abstract

Renal ischemia‐reperfusion (IR) injury and acute kidney injury (AKI) increase the risk of developing hypertension, with T cells suspected as a possible mechanistic link. Endothelin promotes renal T cell infiltration in several diseases, predominantly via the ET_A_ receptor, but its contribution to renal T cell infiltration following renal IR injury is poorly understood. To test whether ET_A_ receptor activation promotes T cell infiltration of the kidney following IR injury, male C57BL/6 mice were treated with the ET_A_ receptor antagonist ABT‐627 or vehicle, commencing 2 days prior to unilateral renal IR injury. Mice were sacrificed at 24 h or 10 days post‐IR for assessment of the initial renal injury and subsequent infiltration of T cells. Vehicle and ABT‐627‐treated mice displayed significant upregulation of endothelin‐1 (ET‐1) in the IR compared to contralateral kidney at both 24 h and 10 days post‐IR (*P *<* *0.001). Renal CD3^+^ T cell numbers were increased in the IR compared to contralateral kidneys at 10 days, but ABT‐627‐treated mice displayed a 35% reduction in this effect in the outer medulla (*P *<* *0.05 vs. vehicle) and a nonsignificant 23% reduction in the cortex compared to vehicle‐treated mice. Whether specific T cell subsets were affected awaits confirmation by flow cytometry, but outer medullary expression of the T helper 17 transcription factor RORγt was reduced by ABT‐627 (*P *=* *0.06). These data indicate that ET‐1 acting via the ET_A_ receptor contributes to renal T cell infiltration post‐IR injury. This may have important implications for immune system‐mediated long‐term consequences of AKI, an area which awaits further investigation.

## Introduction

Ischemic injury to the kidneys as a result of hypoperfusion is a major contributing factor to the development of acute kidney injury (AKI), and to delayed graft function and subsequent allograft deterioration in renal transplant patients. Ischemia‐reperfusion (IR) injury of the kidney induces infiltration by immune cells, which along with resident immune cells contribute to both the injury and repair processes. While much research has focused on the peri‐ischemic period in AKI, there is a growing appreciation that an episode of AKI can have long‐term consequences including increased risk for development of hypertension (Pechman et al. 2009; Hsu et al. 2016) and of transitioning to chronic kidney disease (CKD) (Coca et al. 2012). The mechanisms contributing to these outcomes are an active area of investigation.

T cells play complex roles in the response to renal ischemia. The persistent presence of activated T cells in previously injured kidneys has been proposed by several groups (e.g. Burne‐Taney et al. 2006; Mehrotra et al. 2015) to contribute to long‐term changes in kidney function. Several studies utilizing selective knockout mice and reconstitution with CD4^+^ and/or CD8^+^ T cell subsets implicate CD4^+^ cells in promoting injury during the early phase postischemia (Burne et al. 2001; Day et al. 2006), with these data partially supported by CD4^+^ cell depletion providing protection against injury in some (Pinheiro et al. 2007) but not all (Faubel et al. 2005) studies. Protective or reparative roles for subsets of CD4^+^ T cells have also been identified. Forkhead Box P3 (Foxp3)^+^ T regulatory cells (hereafter referred to as Tregs) increase in injured kidneys at 3 and 10 days following unilateral IR, and depletion or transfer of these cells 24 h after ischemic injury demonstrate that Tregs contribute to repair processes (Gandolfo et al. 2009). A protective anti‐inflammatory role for Tregs mediated by interleukin‐10 (IL‐10) was also described in a bilateral model of renal IR injury (Kinsey et al. 2009). The presence of activated and memory‐effector CD4^+^ and CD8^+^ T cells for weeks after IR injury has also been described (Ascon et al. 2009; Basile et al. 2012). In a rat model of unilateral renal ischemia followed by later contralateral nephrectomy and exposure to a high salt diet, an increase in activated T cells, including IL‐17‐producing T helper 17 (Th17) cells was seen, potentially contributing to the observed induction of salt‐sensitive hypertension (Mehrotra et al. 2015). Treatment of rats with the immune suppressant mycophenolate mofetil had previously been shown to blunt the development of hypertension in that model (Pechman et al. 2008), and more recently angiotensin type 1 receptor blockade was shown to reduce the Th17 infiltration and renal injury in that model (Mehrotra et al. 2015). These data suggest that therapeutic strategies found to either enhance Tregs or decrease Th17 and other injurious T cell subtypes could be beneficial in promoting a healthy outcome following renal IR injury and warrant further investigation.

The peptide endothelin‐1 (ET‐1) is rapidly upregulated in the kidney by ischemia (within 30 min) (Wilhelm et al. 1999) and prolonged elevation (out to 14 days) has been reported in a unilateral renal IR model in mice (Zager et al. 2013). ET‐1, in particular acting via the ET_A_ receptor, has been implicated in inflammatory cell infiltration of the kidney in other disease models including diabetes (Sasser et al. 2007), hypertension (Muller et al. 2000; Boesen et al. 2011), and sickle cell nephropathy (Sabaa et al. 2008; Kasztan et al. 2017). Chronic infusion of a subpressor dose of ET‐1 also promotes upregulation of chemotactic and adhesion molecules, along with inflammatory cell infiltration of the kidney (Saleh et al. 2010). However, only very limited data are available on the effects of ET‐1 on T cell infiltration following renal ischemia, with most previous studies focusing on neutrophils and macrophages (Espinosa et al. 1996; Forbes et al. 1999; Arfian et al. 2012). In an isogeneic rat kidney transplant model, ET_A_ receptor blockade produced an ~50% reduction in the number of T cells present in the kidneys compared to numbers observed in vehicle‐treated rats (Braun et al. 2000). To further investigate the role of endothelin acting via the ET_A_ receptor in renal T cell infiltration following IR injury, the current study tested the effects of ET_A_ receptor blockade on T cell infiltration following 45 min unilateral IR in mice. This unilateral IR model is associated with progression rather than recovery from the IR injury, as seen previously by us (Boesen 2016) and in similar experiments by others (Zager et al. 2011, 2013).

## Materials and Methods

### Animal subjects and tissue collection

Experiments were conducted on 12 week old male C57Bl/6 mice obtained from Jackson Laboratories, Bar Harbor, ME, housed on campus at the University of Nebraska Medical Center (UNMC), Omaha, NE for 1 week prior to use. All procedures were approved in advance by the UNMC Institutional Animal Care and Use Committee. Mice were housed individually and maintained on a normal salt diet (0.3% sodium; Envigo, Madison, WI) throughout the experiment. One treatment group received the highly selective ET_A_ receptor antagonist ABT‐627 (Winn et al. 1996) (“atrasentan”; Abbvie Inc., Abbott Park, IL) in their drinking water (10 mg/kg per day p.o.) throughout the experiment, commencing 2 days prior to surgery, while the other treatment group received normal drinking water, serving as vehicle‐treated controls. A pretreatment rather than interventional treatment strategy was used due to the rapidity with which ET‐1 is upregulated by ischemia (Wilhelm et al. 1999; Boesen 2016).

All mice underwent unilateral renal IR surgery following procedures as described previously (Boesen 2016). Mice were anesthetized with isoflurane (~2% by inhalation; Piramal Enterprises Ltd., Digwal Village, India) and placed in a prone position on a servo‐controlled heating table to maintain core body temperature at 37°C. The right kidney was approached by a retroperitoneal incision and a small microvascular clamp was applied to the renal artery for 45 min, stopping blood flow. The clamp was then removed, allowing reperfusion of the kidney, and 0.4 mL sterile saline was instilled i.p. to replace fluids. The retroperitoneal incision was closed using sterile sutures and surgical staples. Anesthesia was withdrawn and the mouse was allowed to recover, with analgesia provided by buprenorphine (0.1 mg/kg s.c.; Reckitt Benckiser Healthcare (UK) Ltd., Hull, England). Mice were sacrificed under deep isoflurane anesthesia (~5%) either 24 h or 10 days after surgery, with kidneys weighed and either rapidly dissected into cortex, outer, and inner medulla and snap frozen in liquid nitrogen prior to storage at −80°C, or immersion‐fixed in 10% neutral buffered formalin for 24 h and paraffin‐embedded. Plasma was collected by centrifugation of blood obtained by cardiac puncture at 10 days and creatinine concentration measured (Quantichrom Creatinine Assay, BioAssay Systems, Hayward, CA). In a subset of animals at day 10, left ventricles (including septal wall) were dissected and weighed. Numbers of animals per group for frozen tissue and histological analysis cohorts ranged from 6 to 8 for the 24 h time point and 9–12 for the 10 day time point.

### Histological analysis

For T cell quantification, paraffin‐embedded kidneys were sectioned and CD3 immunostaining was performed using a rabbit monoclonal anti‐CD3 antibody (1:50 dilution, Thermo Fisher Scientific, catalogue number MA1‐90582, Rockford IL), with positive staining detected using a Dako EnVision + System‐HRP (DAB) kit (Dako North America, Carpinteria, CA) and slides counterstained with Hematoxylin. T cell numbers were quantified separately in renal cortex and outer medulla by counting CD3^+^ cells in randomly selected nonoverlapping images (20 images for cortex, 10 for outer medulla, up to five for inner medulla) captured at 40× magnification using a Digital Sight DS‐5M‐L1 system (Nikon, Melville, NY) fitted to an Axioskop 20 light microscope (Carl Zeiss, Thornwood, NY) and expressed as average number of positive cells per mm^2^. To assess tubular injury at 24 h post‐IR, tubular profiles within randomly selected, nonoverlapping 40× images (10/cortex, 5/outer medulla) of Periodic acid‐Schiff‐stained IR kidney sections were classified as either normal, dilated, containing casts, or necrotic (including pyknotic or absent nuclei, fragmented epithelium). Renal parenchymal changes after 10 days’ recovery were also assessed using randomly selected, nonoverlapping 40× images (10/cortex, 5/outer medulla) of Periodic acid‐Schiff‐stained IR kidneys. For this analysis, a 10 × 10 grid of points was overlaid on images, and percentage areas occupied by tubules (normal, dilated, cast‐filled or necrotic), vasculature, glomeruli or indeterminate cell types quantified. Animal ID codes which lacked treatment group information were used to identify histological slides. In all cases, brightness and contrast of representative images of were adjusted in an identical fashion in Microsoft Power Point for clarity of presentation.

### Biochemical and molecular analyses

To probe the types of T cells present in the kidneys and potential mechanisms underlying differences between groups, renal tissue was analyzed for selected chemokines, cytokines and characteristic T cell transcription factors. Frozen cortical and outer medullary tissue was homogenized in lysis buffer as described previously (Boesen 2013) and monocyte chemoattractant protein‐1 (MCP‐1) and IL‐17 concentrations in the supernatant determined by ELISA (both from R & D Systems, Minneapolis, MN) and normalized to protein concentration determined by Bradford assay. Total RNA was extracted from frozen tissue using an RNeasy Mini kit (Qiagen, Valencia, CA) and reverse transcribed to cDNA using a QuantiTect Reverse Transcription kit (Qiagen). Expression levels of genes of interest (see below) relative to *β*‐actin were assessed using QuantiTect Primer assays (Qiagen) and Rotor‐Gene SYBR Green PCR kit (Qiagen) according to the manufacturer's recommended cycling conditions on a Rotor‐Gene Q real‐time PCR cycler (Qiagen), and relative fold expression was calculated as 2^−ΔΔCT^ as described previously (Boesen et al. 2012).

### Statistical analysis

Data were analyzed using GraphPad Prism 6 software (v. 6.01). Two‐factor analysis of variance (ANOVA) was used to test for main effects of treatment group (*P*_Treatment_), contralateral versus IR kidney (*P*_Kidney_) and whether treatment group affected the response to IR (*P*_K × T_), together with Bonferroni post hoc tests (corrected for multiple comparisons). Comparisons of single data points between two groups were performed using Student's unpaired *t*‐test or Mann–Whitney *U* test. Data are presented as mean ± SEM unless stated otherwise, with *P *<* *0.05 considered statistically significant.

## Results

### ET_A_ receptor antagonism does not blunt the initial response to renal IR

**Figure 1:**
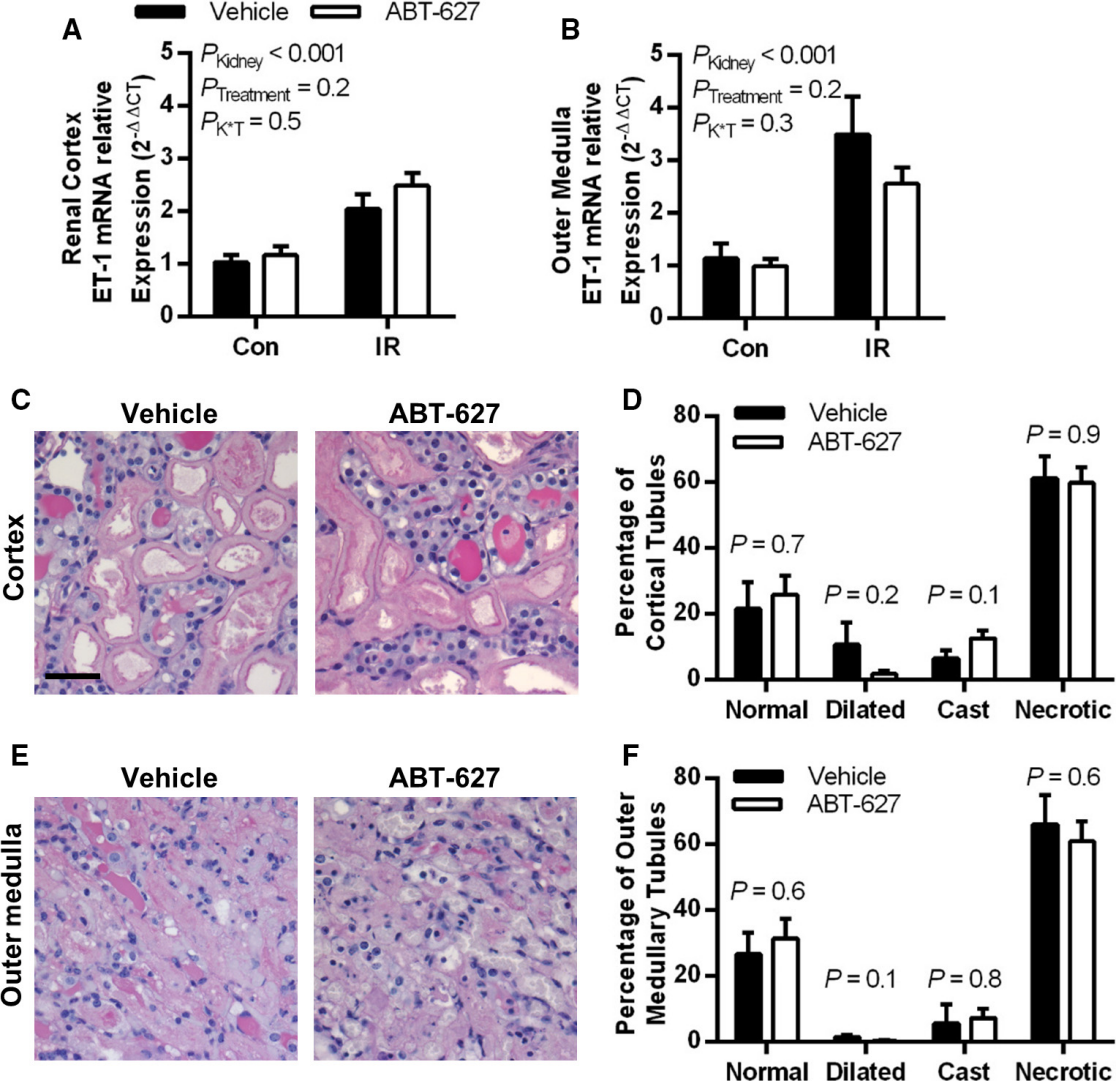
ET‐1 is upregulated by renal IR at 24 h postinjury, with ET_A_ receptor blockade by ABT‐627 providing no attenuation of this response nor of the renal tubular damage observed. ET‐1 in renal cortex (A) and outer medulla (B) was measured by qPCR expressed relative to *β*‐actin and normalized to vehicle‐treated contralateral (Con) kidneys. Data were compared by two‐factor ANOVA, testing for main effects of kidney (*P*_K__idney_), treatment group (*P*_T__reatment_), and the interaction between the two (*P*_K × T_). Periodic acid‐Schiff‐stained kidney sections were evaluated for tubular injury, with (C) depicting representative images of the renal cortex, and (D) showing percentages of tubular profiles categorized as either dilated, cast‐filled, necrotic, or normal. (E) Representative images of the outer medulla, with (F) showing percentages of tubular profiles categorized as either dilated, cast‐filled, necrotic, or normal. *P* values shown in (D) and (F) were determined using Student's unpaired *t*‐test. Black scale bar = 40 *μ*m. Data are presented as mean ± SEM for *n* = 6–8 per group in all cases. ET‐1, endothelin‐1; IR, ischemia‐reperfusion; ANOVA, analysis of variance.

At 24 h post‐IR injury, ET‐1 mRNA was approximately doubled in the cortex of the IR kidney compared to the contralateral kidney (Fig. 1A), and increased further in the outer medulla of the IR kidney (Fig. 1B). Tubular injury in the renal cortex and medulla of the IR kidney was similarly extensive in vehicle and ABT‐627 treated mice, with approximately two‐thirds of tubular profiles showing signs of injury and necrosis (Fig. 1C and D). Kidney weight to body weight ratio was significantly increased by IR (*P*_Kidney_ < 0.001), consistent with tissue swelling and edema, in both vehicle (7.2 ± 0.2 vs. 6.3 ± 0.1 mg/g in IR vs. contralateral) and ABT‐627 treatment groups (7.1 ± 0.2 vs. 6.4 ± 0.1 mg/g in IR vs. contralateral), with no significant difference in this effect between treatment groups (*P*_Treatment_ = 0.9; *P*_K × T_ = 0.5).

**Figure 2:**
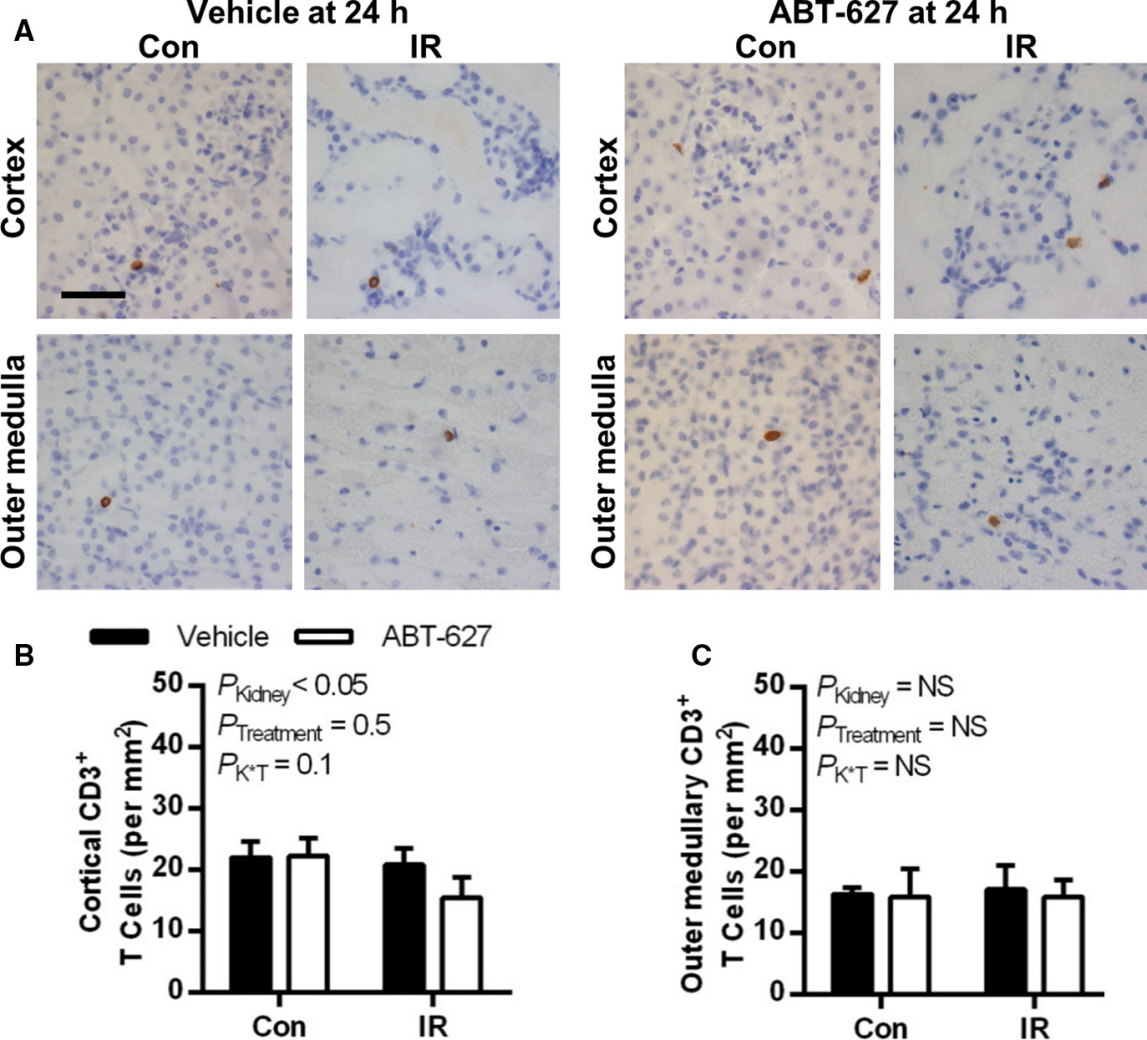
At 24 h post‐IR, renal T cell numbers were not significantly different between vehicle and ET_A_ receptor antagonist treated groups or affected by IR injury. (A) Representative histologic images of CD3^+^ immunostaining (brown pigment) of kidney sections from vehicle and ABT‐627‐treated mice. Quantification of CD3^+^ cells are shown in (B) for the renal cortex and (C) for the outer medulla. Data are presented as mean ± SEM for *n* = 7 per group and were compared by two‐factor ANOVA, testing for main effects of kidney (*P*_K__idney_), treatment group (*P*_T__reatment_), and the interaction between the two (*P*_K × T_). Black scale bar = 40 *μ*m. IR, ischemia‐reperfusion; ANOVA, analysis of variance.

The numbers of CD3^+^ T cells present in the renal cortex and outer medulla of the IR kidney were not significantly elevated compared to the contralateral kidney at 24 h post‐IR injury (Fig. 2). Very few T cells were present in the inner medulla of either group in either kidney, averaging less than 1 CD3^+^ cell per 40× image (data not shown). Despite the lack of influx of T cells at this time point, MCP‐1 was significantly upregulated in the IR compared to contralateral kidney of both groups (*P*_Kidney_ < 0.0001), averaging 64 ± 9 and 77 ± 5 pg/mg in the renal cortex of vehicle and ABT‐627 groups compared with 5 ± 1 and 8 ± 1 pg/mg, respectively, in the contralateral kidney, with no significant difference between treatment groups. High levels of MCP‐1 were found in the outer medulla of the IR kidney in both vehicle (158 ± 12 pg/mg) and ABT‐627 groups (141 ± 10 pg/mg; *P *=* *0.3). MCP‐1 concentrations in the contralateral renal cortex of both groups ranged from <3–12 pg/mg, with no significant difference detected between groups by Mann–Whitney *U*‐test (*P *=* *0.3).

### T cell accumulation in the kidney post‐IR is partially mediated by ET_A_ receptor activation

**Figure 3:**
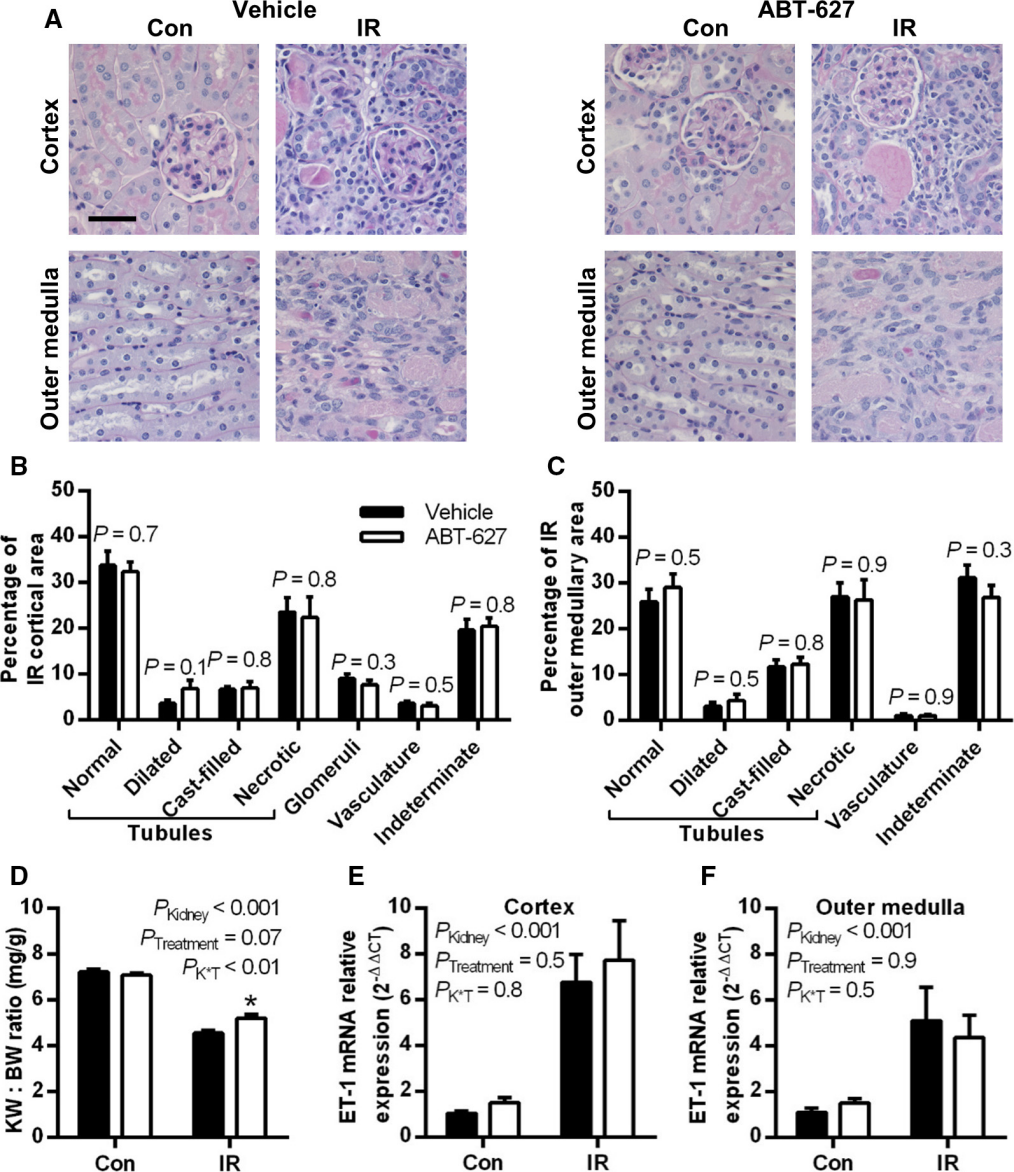
Renal injury, atrophy, and ET‐1 upregulation were observed 10 days post‐IR. (A) Representative images of Periodic‐acid Schiff‐stained kidney sections, with black scale bar = 40 *μ*m. Sections were evaluated for percentage area occupied by different structures, with results shown for renal cortex (B) and outer medulla (C), and data compared between groups by Student's unpaired *t*‐test; *n* = 9–10 per group. (D) Kidney weight to body weight (KW:BW) ratio in contralateral and IR kidneys, with data combined from kidneys collected for histological and molecular analysis (*n* = 20–22 per group). ET‐1 mRNA expression relative to *β*‐actin and normalized to vehicle‐treated contralateral kidneys in (E) cortex and (F) outer medulla of contralateral and IR kidneys (*n* = 10–12 per group). Data for (D–F) are presented as mean ± SEM and compared by two‐factor ANOVA, testing for main effects of kidney (*P*_K__idney_), treatment group (*P*_T__reatment_), and the interaction between the two (*P*_K × T_). ET‐1, endothelin‐1; IR, ischemia‐reperfusion; ANOVA, analysis of variance.

At 10 days post‐IR, substantial parenchymal damage was observed in the cortex and outer medulla of both groups, with evidence of tubular necrosis, cast formation, tubular atrophy, and increased cellularity in the tubulointerstitial space (Fig. 3A). These effects were similar between vehicle and ABT‐627 treated groups in both the cortex (Fig. 3B) and outer medulla (Fig. 3C). Kidney to body weight ratio was significantly reduced in the IR compared with contralateral kidneys in both groups (*P*_Kidney_ < 0.001), however, ABT‐627 treatment slightly but significantly attenuated the relative reduction in IR kidney mass compared with the vehicle group (*P *<* *0.05; Fig. 3D). In both treatment groups, ET‐1 mRNA levels remained dramatically increased in the IR compared to contralateral renal cortex and outer medulla (Figs. 3E and F; *P*_Kidney_ < 0.001 in both regions). Plasma creatinine concentration was within the normal range for mice and was not significantly different between groups (0.341 ± 0.014 mg/dL in vehicle and 0.344 ± 0.015 mg/dL in ABT‐627‐treated mice, *n* = 20 and 22; *P *=* *0.9). Left ventricle to body weight ratio was measured in a subset of mice in each group (*n* = 7–8), but this was also within the normal range and not significantly different between groups (3.42 ± 0.06 and 3.53 ± 0.17 mg/g in vehicle and ABT‐627 groups, respectively, *P *=* *0.6).

**Figure 4:**
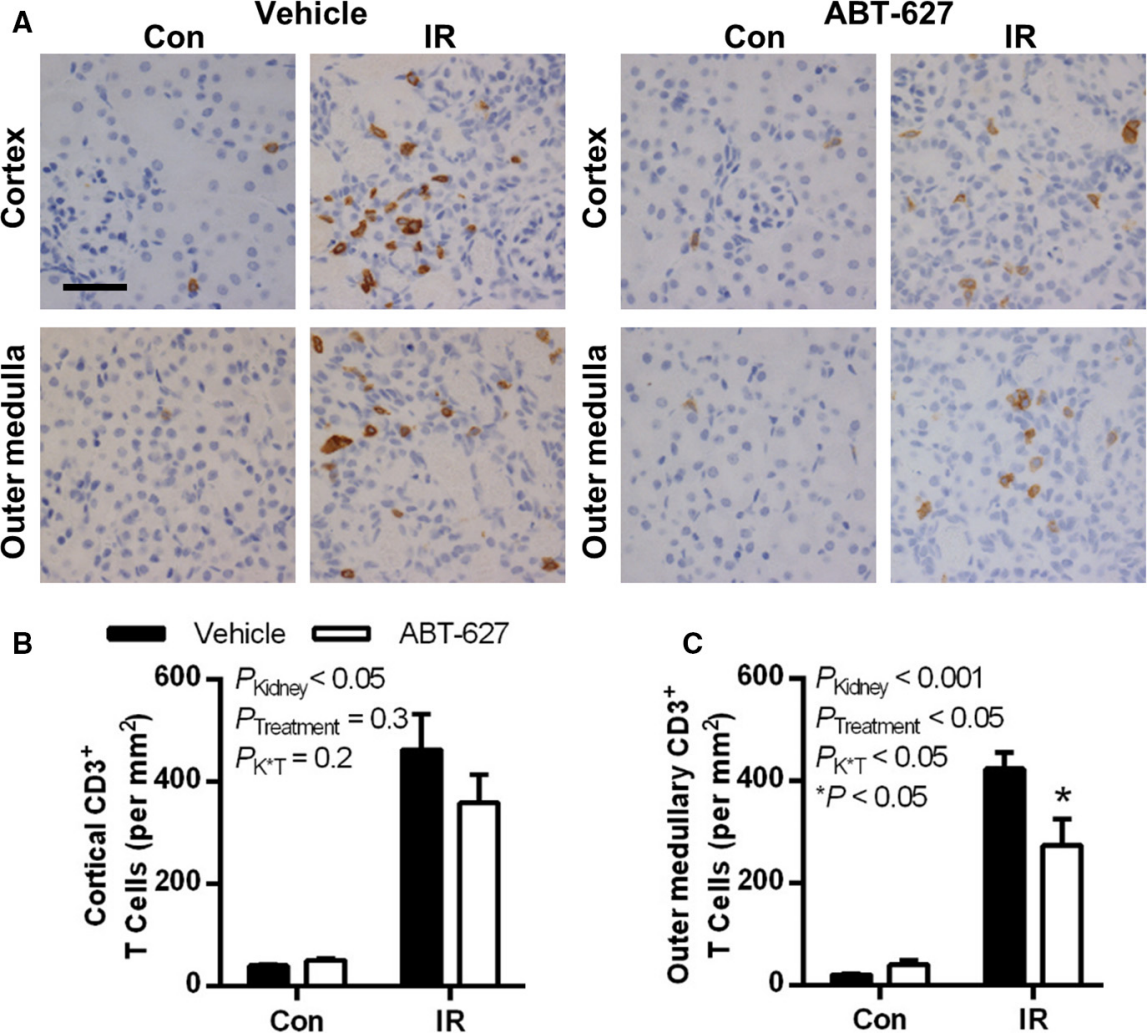
At 10 days post‐IR, renal T cell numbers were significantly increased in the IR kidney compared to the contralateral kidney, an effect significantly attenuated in the outer medulla by ET_A_ receptor blockade. (A) Representative histologic images of CD3^+^ immunostaining (brown pigment) of kidney sections from vehicle and ABT‐627‐treated mice. Quantification of CD3^+^ cells are shown in (B) for the renal cortex and (C) for the outer medulla. Data are presented as mean ± SEM for *n* = 9–10 per group and were compared by two‐factor ANOVA, testing for main effects of kidney (*P*_K__idney_), treatment group (*P*_T__reatment_) and the interaction between the two (*P*_K × T_). *P <* *0.05 versus vehicle group for the same kidney by Bonferroni post hoc test. Black scale bar = 40 *μ*m. IR, ischemia‐reperfusion; ANOVA, analysis of variance.

T cell numbers were dramatically increased in the cortex and outer medulla of the IR kidney at 10 days post‐IR in both treatment groups (*P*_Kidney_ < 0.05; Fig. 4). Treatment with ABT‐627 did not significantly blunt the IR‐induced increase in T cells in the renal cortex (*P*_K × T_ = 0.2; Fig. 4B), but significantly attenuated the increase in T cells in the outer medulla (*P *<* *0.05; Fig. 4C). Very few T cells were present in the inner medulla of either group in either kidney, averaging from 0 to 3 CD3^+^ cells per 40× image (data not shown).

**Figure 5:**
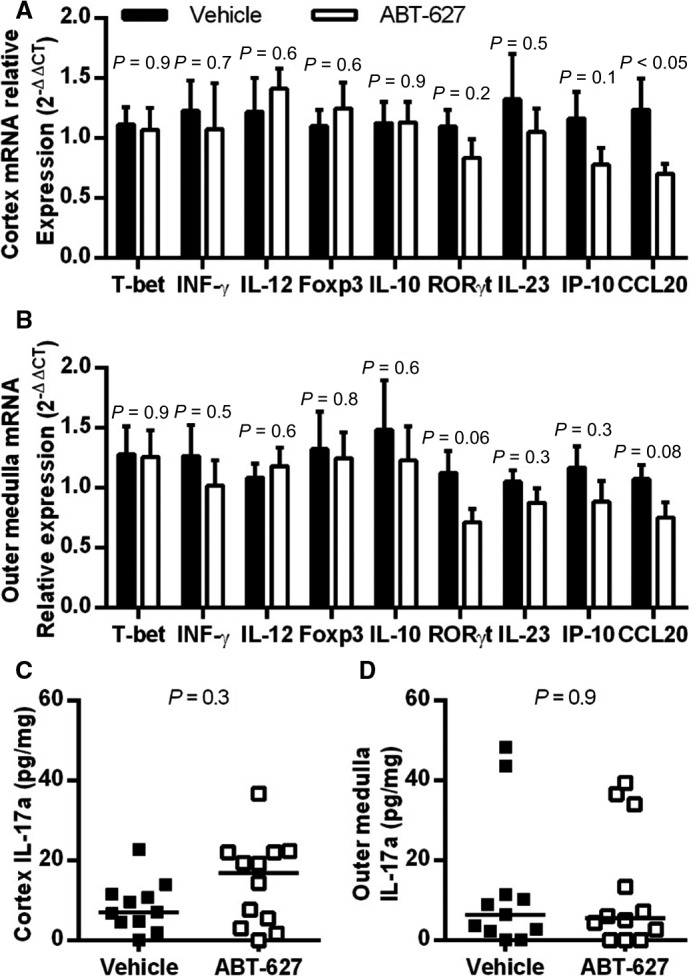
Expression of T cell markers and related cytokines/chemokines at 10 days post‐IR. Expression at the mRNA level, relative to *β*‐actin and normalized to vehicle‐treated IR kidneys is shown for (A) renal cortex and (B) outer medulla (data are mean ± SEM for *n* = 10–12 per group, and *P* values determined by Student's unpaired *t*‐test). Tissue homogenate IL‐17a was measured by ELISA and normalized to protein concentration, with individual data points and median values shown for (C) cortex and (D) outer medulla. Values were below detection in one sample per group for cortex, and in two vehicle and three ABT‐627 outer medulla samples, with these designated as zero for display purposes and for analysis by nonparametric Mann–Whitney *U* test. IR, ischemia‐reperfusion; IL, interleukin.

To further characterize the effect of ABT‐627 on the types of T cells present in the kidneys at 10 days post‐IR, expression levels of select transcription factors and cytokines were compared between treatment groups in IR kidney cortical and outer medullary tissue. There were no differences between groups in expression of the Th1 transcription factor T‐bet (also known as Tbx21), the Th1‐derived cytokine INF‐*γ* or the Th1 polarizing factor IL‐12 (IL‐12a/p35 subunit) in either the cortex or outer medulla (Fig. 5A and B). Expression of the Treg transcription factor Foxp3, and the anti‐inflammatory cytokine IL‐10 were not significantly different between groups in either region (Fig. 5A and B). Th17 transcription factor RAR Related Orphan Receptor (ROR)γt (*RORC* gene) expression was also not significantly different between groups in the renal cortex (Fig. 5A), but was reduced by ABT‐627 in the outer medulla (*P *=* *0.06; Fig. 5B), suggesting that ABT‐627 treatment may reduce Th17 cell infiltration in the outer medulla. IL‐17a mRNA expression was analyzed to further confirm this observation, but expression was very low, with C_T_ values in the high 30 sec and thus considered unreliable for quantification (data not shown). Concentrations of IL‐17a measured in tissue homogenates were also very low, and not significantly different between vehicle and ABT‐627‐treated groups (Fig. 5C and D). IL‐23, which can contribute to polarization of Th17 cells, was not significantly different between groups (Fig. 5A and B). Levels of the Th1‐attracting chemokine IP‐10 (also known as CXCL10) was not significantly different between groups (Fig. 5A and B). CCL20, which can be produced in response to IL‐17 and further attract Th17 cells, was significantly reduced by ABT‐627 treatment in the renal cortex (*P *<* *0.05; Fig. 5A), but this trend did not reach statistical significance in the outer medulla (*P *=* *0.08; Fig. 5B). MCP‐1 concentrations were similar between groups in IR kidney cortexes (269 ± 32 and 273 pg/mg protein in vehicle and ABT‐627 groups, respectively; *P *=* *0.9) and outer medullas (328 ± 29 and 275 ± 26 pg/mg protein in vehicle and ABT‐627 groups, respectively; *P *=* *0.2).

## Discussion

Although much research on renal IR injury, including the role of T cells, has historically focused on the initial 24–72 h post‐IR, the growing recognition of AKI as a risk factor for later development of hypertension and chronic kidney disease (CKD) (Pechman et al. 2009; Coca et al. 2012; Hsu et al. 2016) makes more long‐term analysis of the role of T cells following IR injury a relevant area of inquiry. The current study provides evidence that the renal T cell infiltration seen at 10 days post‐IR injury is partially dependent on ET‐1 acting via the ET_A_ receptor, particularly in the outer medulla. These findings occurred despite a similar degree of renal injury being observed at 24 h post‐IR, as determined from histological scoring of renal tubular morphology. Although not definitive, the lack of a difference in left ventricle to bodyweight ratio between groups suggests that this reduction in outer medullary T cells by ABT‐627 treatment was not due to amelioration of hypertension, in contrast to what was seen previously in the Angiotensin II‐induced hypertension model in mice (Boesen et al. 2011). Additional novel data in the current study showed that ET_A_ receptor blockade reduced expression of the transcription factor RORγt, a marker of Th17 cells, and a reduction in CCL20, which can attract Th17 cells (Bromley et al. 2008). However, IL‐17a concentrations in tissue homogenates did not mirror this finding; renal tubular cells can also produce IL‐17 (Norlander et al. 2016), perhaps masking any difference in the immune cell contribution. While provocative, our data indicate a need for further investigation to resolve whether ET_A_ receptor activation preferentially stimulates recruitment of Th17 cells or affects multiple T cell subsets.

The ability of ET_A_ receptor antagonism to reduce T cell accumulation in the kidney following IR injury agrees well with data from several other disease models, including renal transplant (Braun et al. 2000), hypertension (Boesen et al. 2011), and sickle cell disease (Kasztan et al. 2017). Together, the reduction in total CD3^+^ T cell numbers and RORγt expression in the outer medulla of ABT‐627‐treated mice observed in the current study could suggest that ET_A_ receptor blockade reduces the differentiation and/or infiltration of T cells, possibly with a preferential effect on Th17 cells, following IR injury. In vitro experiments suggest that ET‐1 does not directly influence T cell differentiation, as coincubation of naive CD4^+^ T cells with ET‐1 did not alter their production of cytokines typical of Th1 (INF‐*γ*), Th2 (IL‐4), or Th17 cells (IL‐17), nor did it affect Foxp3 expression (Tajiri et al. 2014). Our data are consistent with several of these measurements, although we were unable to test whether Th2 cells were affected, as the main marker used to characterize these cells, GATA3, is also expressed by renal epithelial cells (Miettinen et al. 2014). In a separate study (Tanaka et al. 2014), ET_A_ receptor blockade did not blunt the increase in RORγt mRNA expression in response to exposure of naive T cells to Th17‐polarizing agents, but did reduce IL‐17 production. We further found no difference between groups in mRNA levels of IL‐12 (Th1‐polarizing) or IL‐23 (Th17‐polarizing). Accordingly, the mechanism by which ET_A_ receptor blockade might influence the balance of different types of T cells is currently unclear. To the author's knowledge, studies to date have not further characterized the effect of ET‐1 on renal T cell subpopulations in any disease state by flow cytometry, which is a worthy area of future investigation but was beyond the scope of the current study.

IL‐17 has been shown to promote fibrosis and infiltration of immune cells in renal IR injury (Mehrotra et al. 2017), and has been implicated in alterations in tubular sodium handling and the development of hypertension in response to chronic angiotensin II infusion (Madhur et al. 2010; Norlander et al. 2016), making it an attractive target in terms of understanding the mechanisms involved in the development of hypertension post‐AKI and the increased risk of progression to CKD. However, no significant difference in IL‐17a expression was detected between groups in the present study, which was somewhat surprising given several lines of evidence suggesting that ET‐1 can stimulate IL‐17 production by Th17 or possibly other cell types. As mentioned above, ET_A_ receptor blockade attenuated IL‐17 production following activation of differentiated Th17 cells in vitro (Tanaka et al. 2014). Transgenic overexpression of ET‐1 in either the endothelium or by astrocytes enhances splenic lymphocyte IL‐17a production and exacerbates the rise in serum IL‐17 levels in experimental allergic encephalomyelitis (Guo et al. 2014). ET_A_ receptor blockade reduced circulating IL‐17 levels in a rodent model of hemolysis‐elevated liver enzymes‐low platelets syndrome in conjunction with reducing numbers of circulating CD4^+^ T cells (Morris et al. 2016). Interestingly, Cornelius et al. (2013) demonstrated that IL‐17 infusion had an inhibitory effect on ET‐1 production in renal cortex of pregnant female rats but no effect on human umbilical vein endothelial cell ET‐1 production, suggesting a complex relationship between the two substances. Natural Killer cells were also recently identified as a potential source of IL‐17 in post‐IR kidneys (Mehrotra et al. 2017), and potential nonimmune sources of IL‐17 may include renal epithelial cells (Norlander et al. 2016). Accordingly, it remains possible that any effect of ET_A_ receptor blockade to reduce T cell production of IL‐17 in the current study was masked by IL‐17 production by other cell types. The relative contributions of tubular epithelial and immune cell sources of IL‐17 in renal IR injury await further investigation.

The injury induced by 45 min unilateral renal IR is relatively severe, and ET_A_ receptor blockade does not appreciably reduce histological evidence of renal injury out to 28 days post‐IR in this model (Boesen 2016). Those findings stand in contrast to another study by Zager et al. (2013), who used a shorter duration of ischemia (30 min) and a different mouse genetic background (CD‐1), reporting that renal injury was markedly attenuated by ET_A_ receptor blockade at 14 days post‐IR. T cell infiltration was not measured in Zager et al. (2013) study. A serendipitous advantage of the model used in the current study is that since histological renal injury at 24 h was similar in both groups, the reduction in T cells in the ET_A_ receptor antagonist group seen at 10 days is unlikely to be attributable to a diminution of the severity of the initial phase of renal injury. The mechanism underlying the statistically significant effect of ET_A_ receptor blockade on outer medullary but not cortical T cell numbers is not readily apparent from the current data, despite our assessment of histological injury, cytokine, and chemokine expression.

Together with earlier findings in a renal transplant model (Braun et al. 2000), these results indicate that ET_A_ receptor blockade can attenuate T cell infiltration of the kidney post‐IR, particularly in the outer medulla. Future studies using flow cytometry to determine the impact of ET_A_ receptor blockade on renal T cell subsets post‐IR injury and in other models of renal disease are also warranted.

## Conflict of Interest

The author has previously received research funding from Abbvie, Inc. for a separate series of studies investigating the role of endothelin in acute renal failure.

## Data Accessibility

Data that underlie conclusions reported in this article will be made available to readers on reasonable request.
